# Nanoscale Thermal Cloaking in Silicon Film: A Molecular Dynamic Study

**DOI:** 10.3390/ma15030935

**Published:** 2022-01-26

**Authors:** Jian Zhang, Haochun Zhang, Wenbo Sun, Qi Wang, Dong Zhang

**Affiliations:** Harbin Institute of Technology, School of Energy and Engineering, Harbin 150001, China; 20B902063@stu.hit.edu.cn (J.Z.); 21S102109@stu.hit.edu.cn (W.S.); 21S002039@stu.hit.edu.cn (Q.W.); 20S002016@stu.hit.edu.cn (D.Z.)

**Keywords:** nanoscale, silicon, thermal cloak, dynamic response, molecular dynamics

## Abstract

Nanoscale thermal shielding is becoming increasingly important with the miniaturization of microelectronic devices. They have important uses in the field of thermal design to isolate electronic components. Several nanoscale thermal cloaks based on graphene and crystalline silicon films have been designed and experimentally verified. No study has been found that simultaneously treats the functional region of thermal cloak by amorphization and perforation methods. Therefore, in this paper, we construct a thermal cloak by the above methods, and the ratio of thermal cloaking and response temperature is used to explore its cloaking performance under constant and dynamic temperature boundary. We find that compared with the dynamic boundary, the cloaking effect produced under the constant boundary is more obvious. Under two temperature boundaries, the thermal cloak composed of amorphous and perforated has a better performance and has the least disturbance to the background temperature field. The phonon localization effect produced by the amorphous structure is more obvious than that of the perforated structure. The phonon localization of the functional region is the main reason for the cloaking phenomenon, and the stronger the phonon localization, the lower the thermal conductivity and the more obvious the cloaking effect. Our study extends the nanoscale thermal cloak construction method and facilitates the development of other nanoscale thermal functional devices.

## 1. Introduction

The “invisibility cloak” that appears in fiction has always attracted the interest of researchers. With the emergence of the theory of transformation optics [1], researchers successfully designed an optical thermal cloak and experimentally verified it with the help of metamaterials. Subsequently, Fan et al. [2] introduced the above theory into the field of thermotics and predicted thermal cloaks. With the emergence of transformation thermotics and thermal metamaterials, the thermal cloak greatly attracted the interest of researchers. Based on the theory of transformation thermotics, researchers have constructed a number of varieties of thermal functional devices: for instance, thermal cloak [3,4,5,6,7,8,9,10,11,12], thermal concentrator [13,14], thermal illusion [15,16,17,18], thermal camouflage [19,20,21,22,23,24,25], thermal rotator [26], and encrypted thermal printing [27]. The thermal cloak, as a typical representative of heat flux control devices, was first experimentally verified [28]. However, the thermal cloak with a perfect cloaking effect cannot be prepared yet due to the singularity characteristics generated by transformation thermotics. The theory of transformation thermotics mainly focuses on the heat conduction equation, and there is a relative lack of research involving thermal convection and thermal radiation.

At the nanoscale, due to the failure of Fourier’s law of heat conduction, researchers have designed a variety of nanodevices with the help of molecular dynamics tools, such as thermal diodes [29,30], thermal rectification [31,32,33,34], topological phonon Hall effect [35,36,37], etc. With the rapid development of packaging electronics, various electronic devices show the development trend of miniaturization and integration, resulting in a significant increase in the heat generated when the electronic equipment is working. Therefore, the electronic equipment must be reasonably arranged to ensure the normal operation of the electronic equipment. The most effective method is to thermally shield the heat-sensitive components near the heat source, which can be achieved through physical isolation or thermal insulation. Therefore, studying the design and mechanism analysis of the nanoscale thermal cloak has important engineering significance. Ye et al. [38] used graphene to build a chemically functionalized thermal cloak. Liu et al. [39] used the “melting-quenching” technique to amorphize crystalline silicon and successfully build a thermal cloak. Choe et al. [40] successfully observed the cloaking phenomenon on the nanoscale by designing an ion irradiation platform. Hu et al. [41] introduced how to use two-dimensional phonon engineering to explore possible microscale thermal functions. However, a combination of amorphization and perforation has not been found yet. Therefore, we built nanoscale thermal cloaks by this method for analysis. 

By the combination of amorphization and perforation, we constructed a nanoscale thermal cloak. Moreover, to explore its cloaking performance, we built an amorphized thermal cloak and a perforated thermal cloak and a crystalline silicon film as a comparison. The cloaking performance was first explored by the ratio of thermal cloaking, and the heat flux and temperature distribution were plotted by selecting the best cloaking structure. Then, we calculated the response temperature to study the disturbance to the temperature field in the background region with the existence of the cloak. Finally, using phonon localization theory, the mechanism was explained by calculating the PDOS and MPR. Our study extends the nanoscale thermal cloak construction method, which can promote its engineering application.

## 2. Model and Methodology

**Figure 1:**
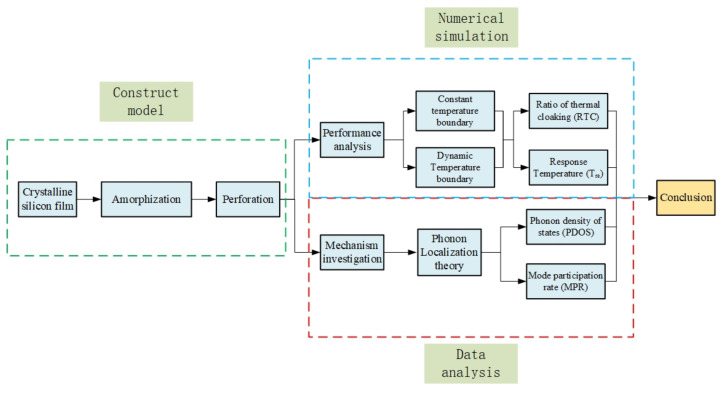
The calculation flow chart.

In this section, we first construct the computational model, then introduce the basic theory, and finally describe the computational process, which is shown in Figure 1.

### 2.1. Model Structure

**Figure 2:**
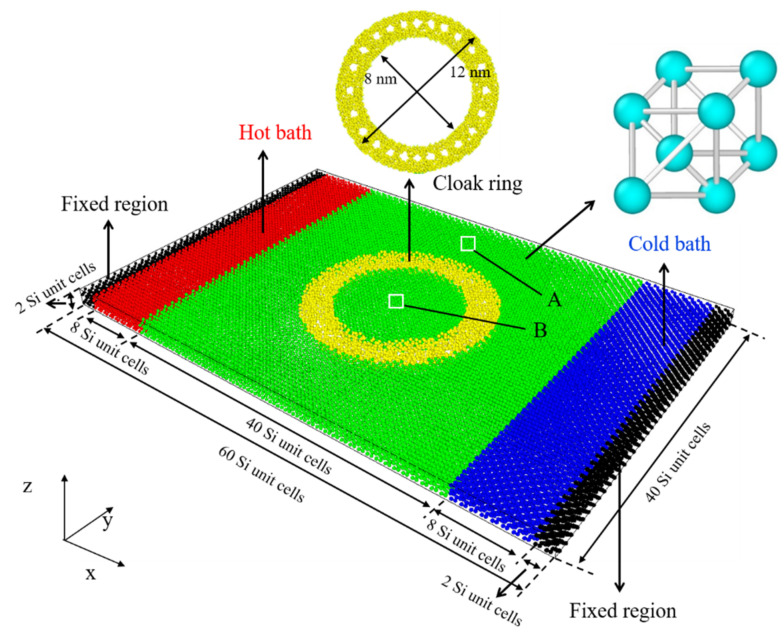
Model of the nanoscale thermal cloak.

As shown in Figure 2, based on our previous study, we establish the nanoscale thermal cloak. This study adopts the built-in modeling command of LAMMPS for modeling because Si is a diamond structure. The lattice constant of Si is 5.431 Å, and the size of the simulation box is 60 × 40 × 2 Si unite cells (UC). The whole system is divided into two parts: the boundary and the calculated domain. The boundary consists of a fixed and a thermostat region, and the calculated domain includes a functional and a background region. The fixed region is located in the two ends of the system to prevent the whole system moving, and its length along the x-direction is 2 UC. The thermostat region consists of a hot bath and a cold bath, both of which are 8 UC along the x-direction. The functional region is constructed by amorphization and perforation, and the entire region is amorphous silicon.

### 2.2. Methodology

In this study, the velocity Verlet algorithm [42] was adopted to numerically integrate the Newton’s equation of motion with MD. Canonical ensemble (NVT) and microcanonical ensemble (NVE) were adopted for the dynamic simulation.

#### 2.2.1. Potential Energy Model

The potential function was used to describe the interaction between atoms, and the correctness of the calculation results also depended on the choice of the potential function; we chose the most commonly used Tersoff potential for the calculation [43]:

(1) $$E=\sum_{i}E_{i}=\frac{1}{2}\sum_{i\neq j}V_{ij}\;V_{ij}=f_{C}\left(r_{ij}\right)\left[f_{R}\left(r_{ij}\right)+b_{ij}f_{A}\left(r_{ij}\right)\right]$$

where *E* is the system energy and *i* and *j* are the atomic number. *r* is the atomic distance. *f* is the function, *f_R_* is the repellent potential, *f_A_* is the attractive potential, *f_C_* is the cutoff potential, and *b* is atomic bond order.

#### 2.2.2. Ratio of Thermal Cloaking (*RTC*)

During the simulation, we calculated the single-atom heat flux:

(2) $$J=\frac{1}{V}\left[\sum_{i}e_{i}v_{i}-\sum_{i}S_{i}v_{i}\right]$$

where J is the heat flux, *V* is the volume, and *e* is the total energy. **v** is the atomic velocity vector. **S** is the atomic pressure tensor. The heat flux in three directions can be written as:

(3) $$J_{x}=\frac{1}{V}\left[\sum_{i}e_{i}v_{xi}-\sum_{i}\left(S_{ixx}v_{ix}+S_{ixy}v_{iy}+S_{ixz}v_{iz}\right)\right]\;J_{y}=\frac{1}{V}\left[\sum_{i}e_{i}v_{yi}-\sum_{i}\left(S_{iyx}v_{ix}+S_{iyy}v_{iy}+S_{iyz}v_{iz}\right)\right]\;J_{z}=\frac{1}{V}\left[\sum_{i}e_{i}v_{zi}-\sum_{i}\left(S_{izx}v_{ix}+S_{izy}v_{iy}+S_{izz}v_{iz}\right)\right]$$

As shown in Figure 1, we calculated the single-atom heat flux in the A and B regions of the different structures and further calculated the average heat flux for the purpose of analysis. The ratio of thermal cloaking (*RTC*) was used to calculate the cloaking performance of the thermal cloak, which can be expressed as:

(4) $$RTC=\frac{J_{A}}{J_{B}}$$

where *J_A_* represents the average heat flux in region *A*, while *J_B_* represents the average heat flux in region *B*.

#### 2.2.3. Response Temperature (*T_re_*)

Temperature is a macroscopic thermophysical property and an important index to evaluate the cloaking effect. Therefore, during the simulation, we calculated the single-atom temperature:

(5) $$\sum_{i}\varepsilon_{i}=\frac{dim}{2}k_{B}NT$$

where *i* is the atomic lable, *ԑ* stands for the kinetic energy, *dim* denotes the dimensionality, here *dim* = 3, *N* is the total atomic number in the calculation region, and *k_B_* is the Boltzmann constant.

We calculated the average temperature of the selected regions and further analyzed the influence of the thermal cloak on the temperature field in the background region through the response temperature (*T_re_*):

(6) $$T_{re}=\frac{\Delta T_{A}}{\Delta T_{B}}\;\Delta T_{A}=\left|T_{A}^{'}-T_{A}\right|\;\Delta T_{B}=\left|T_{B}^{'}-T_{B}\right|$$

where *T_A_* represents the average temperature of the *A* region in the crystalline film, *T_A_’* represents average temperature of the *A* region in the film with cloak, *T_B_* represents the average temperature of the *B* region in the crystalline film, and *T_B_’* represents average temperature of the *B* region in the film with cloak.

#### 2.2.4. Phonon Density of States (PDOS)

To understand the working mechanism, we first calculated the phonon density of states (PDOS). Moreover, for the purpose of warranting the same region variables, we picked the same calculated region in three structures, and we took the 2 nm ring containing all functional regions as the calculated domain. We could obtain PDOS by the Fourier transform of the velocity autocorrelation function:

(7) $$\mathrm{PDOS}\left(\omega \right)=\frac{1}{N\sqrt{2\pi }}\int e^{-i\omega t}\langle \sum_{j=1}^{N}v_{j}\left(0\right)v_{j}\left(t\right)\rangle dt$$

where *N* is the total atomic number in the calculated region, *ω* is the phonon frequency, and $v_{j}$ denotes the velocity vector of the *j*th atom.

#### 2.2.5. Mode Participation Ratio (MPR)

To better explain the cloaking phenomenon, we calculated the mode participation rate (MPR) [44,45]:

(8) $$\mathrm{MPR}\left(\omega \right)=\frac{1}{N}\frac{{\left[\sum_{i}{\mathrm{PDOS}}_{i}{\left(\omega \right)}^{2}\right]}^{2}}{{\sum_{i}{\mathrm{PDOS}}_{i}\left(\omega \right)}^{4}}$$

where *N* represents the total number of atoms in the calculated domain, and according to Equation (7), PDOS*_i_* (*ω*) is the local density of states.

### 2.3. Simulation Process

In this study, large-scale atomic/molecular massively parallel simulator (LAMMPS) [46] software was used to simulate the nonequilibrium molecular dynamics (NEMD) process. The simulated data were visualized and analyzed on Ovito [47] software. The Nose-Hoover thermostat was used in the simulation to keep the temperature of the system constant [48]:

(9) $$\frac{d}{dt}p_{i}=F_{i}-\gamma p_{i}$$

(10) $$\frac{d}{dt}\gamma =\frac{1}{\tau^{2}}\left[\frac{T\left(t\right)}{T_{0}}-1\right]$$

(11) $$T\left(t\right)=\frac{2}{3Nk_{B}}\sum_{i}\frac{p_{i}^{2}}{2m_{i}}$$

where *i* is the atomic label, *p* is the momentum, *m* is the mass, and *F* is the force. *γ* is the dynamic parameters, *τ* is the relaxation times, *k_B_* is the Boltzmann constant, and *N* is the total atomic number of the thermostats. 

**Figure 3:**
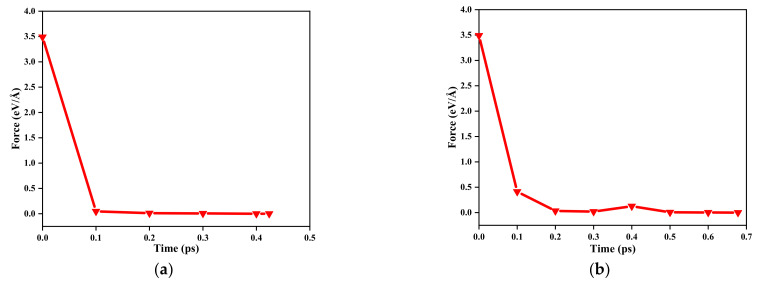
Energy minimization process of different structures: (**a**) amorphous; (**b**) amorphous + perforated.

**Figure 4:**
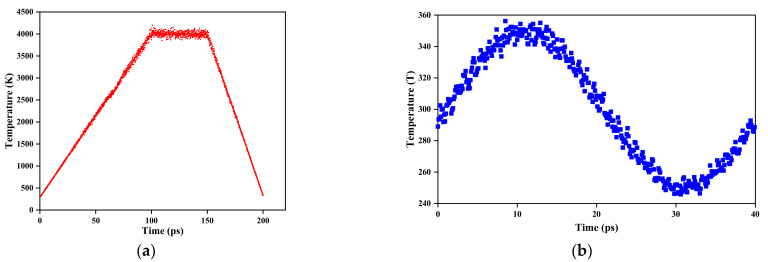
(**a**) Temperature variation during annealing in the cloaking region; (**b**) dynamic temperature boundary.

Periodic boundary conditions are applied to all three directions of the system. The time step is 1 fs for all simulations. First, the simulated annealing process. According to Reference [49], amorphization of crystalline silicon is performed. To minimize the energy of the model, it was stopped when all the components of force on atoms less than 10**^−^**^3^ eV**/**Å. The energy minimization processes of different structures are shown in Figure 3. Fix the atoms in the fixed region and give other atoms an initial velocity corresponding the values at 300 K, and these velocities obey the Gaussian distribution. Relax the whole system except for the fixed region in the NVT ensemble for 1000 ps. Afterward, at the rate of 3.7 × 10^13^ K/s, the functional region was heated from 300 to 4000 K and maintained for 50 ps at 4000 K, so that the crystalline silicon can melt. Next, the temperature was quickly quenched to 300 K, and the functional region was transformed from crystalline to amorphous. During the process, the temperature variation of the functional region is shown in Figure 4a. Second, the NEMD process. Minimize energy again and then give all atoms except the atoms in the fixed region an initial velocity. Then, in the NVT ensemble, the system was equilibrated for 100 ps at 300 K. The cold bath was fixed at 250 K, while the hot bath adopts two kinds of boundaries, the constant was 350 K or the dynamic temperature boundary which is shown in Figure 4b. In the NVE ensemble, the system, except for the fixed and thermostat regions, was simulated for 1000 ps.

## 3. Results and Discussions

### 3.1. The Cloaking Performance

**Figure 5:**
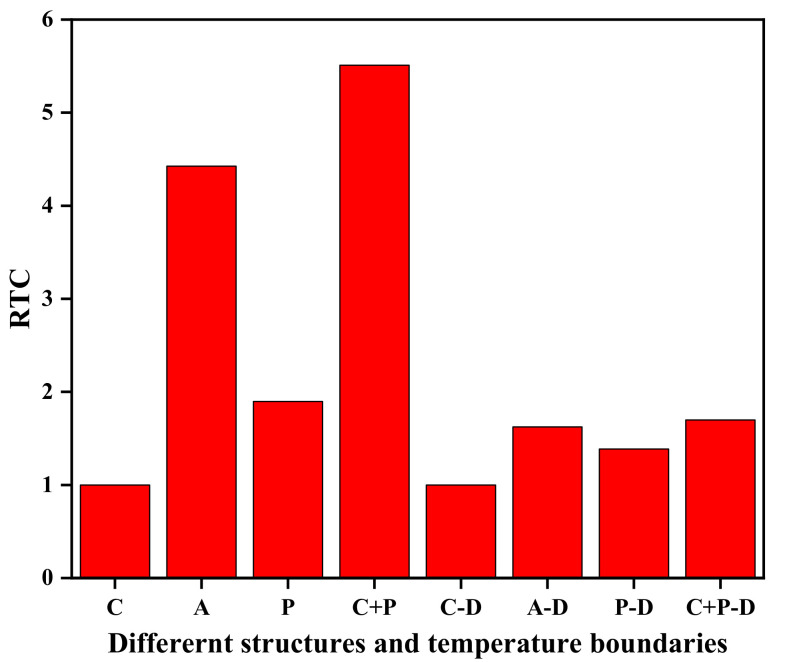
*RTC* at different structures and temperature boundaries, where C denotes crystal, A denotes amorphous, P denotes perforated, and D denotes dynamic.

Figure 5 shows the *RTC* of different structures and temperature boundaries at 400 ps. For the crystalline silicon film, at different temperature boundaries, the *RTC* is all 1, while the *RTC* of the silicon film with a cloak is greater than 1, which proves that the phenomenon of cloaking has occurred. At different temperature boundaries, the *RTC* of the amorphous and perforated silicon film is the largest, and the *RTC* of the perforated silicon film is the smallest. To facilitate the perforation, the model we build has a larger functional region, and no identical models have been found, but the trend presented by *RTC* is consistent with previous research [39]. In addition, compared to the dynamic temperature boundary, the cloaking effect produced under the constant temperature boundary is more obvious. The reason is that the average temperature of the dynamic boundary at the same time is much lower than the temperature of the constant boundary, and the heat flux transferred in the whole system is small, resulting in an insignificant cloaking effect.

**Figure 6:**
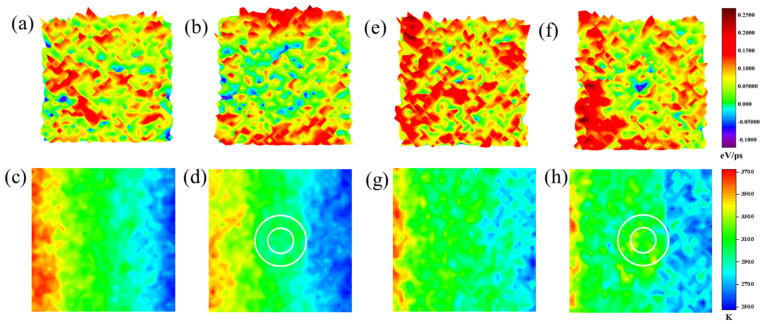
The heat flux and temperature distribution of different structures and temperature boundaries at 400 ps: (**a**,**b**) the heat flux distribution of the perfect film, amorphous, and perforated film at constant temperature boundary, respectively; (**c**,**d**) the temperature distribution of the perfect film, amorphous and perforated film at constant temperature boundary, respectively; (**e**,**f**) the heat flux distribution of the perfect film, amorphous, and perforated film at dynamic temperature boundary, respectively; (**g**,**h**) the temperature distribution of the perfect film, amorphous, and perforated film at constant temperature boundary, respectively.

We have drawn the temperature and heat flux distribution of the crystalline film and the best cloaking structure to show the cloaking phenomenon. Referring to Reference [50], the single-atom heat flux was calculated. Since temperature and heat flux are macroscopic quantities, we divided the system except for the fixed and the thermostat regions into 40 × 40 small blocks, calculated the average temperature and heat flux of each small block, and drew the distribution map, as shown in Figure 6. Due to the existence of the cloak, the heat flux bypasses the functional region, realizing the regulation of the heat flux. Meanwhile, in temperature distribution, the temperature near the cloak has a significant change compared with that of the crystalline silicon film, indicating that the existence of the cloak does adjust the heat transfer. However, the temperature of the functional region is not kept at a low temperature, and there is no obvious cloaking phenomenon. This is because the unique function of the thermal cloak is to control the heat flux, rather than keeping the cloaking region at a low temperature.

We further calculate the average temperature of the selected regions and analyze the influence of the thermal cloak on the external temperature field through the response temperature (*T_re_*), as shown in Table 1. The response temperature of the perforated silicon film is the largest, and the response temperature of the amorphous and perforated silicon film is the smallest. Moreover, for different structures, the constant temperature boundary has a smaller response temperature than the dynamic temperature boundary. The conclusion is consistent with the *RTC* evaluation index. For different temperature boundaries, the thermal cloak constructed by amorphous and perforation can produce the best cloaking performance, and at this time, the interference to the external temperature field is minimal.

### 3.2. The Cloaking Mechanism

**Figure 7:**
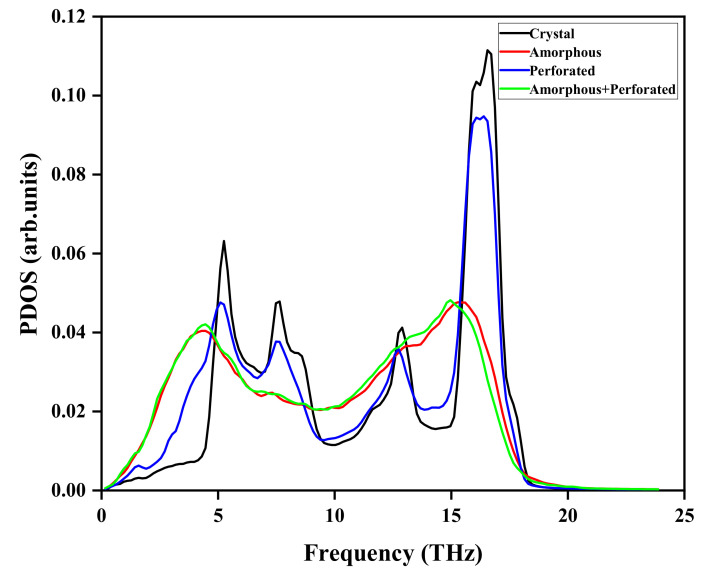
PDOS of different structures.

Figure 7 shows the PDOS of different structures. The PDOS of the perforated film and the crystalline film are close, while the PDOS of the amorphous film and amorphous perforated film are close. For the crystalline film, in accordance with previous studies [51], there is a strong peak at high frequency of 17 THz and a weak peak at low frequency of 5 THz. For the perforated silicon film, the reduction of atoms in the perforated region leads to a decrease in the peak and a shift to the left. As for the amorphous silicon film, because the vibration frequency of the amorphous silicon atoms is lower, the peak at high frequency is weaker while the modulus at low frequency increases. Moreover, the amorphous silicon film shows a large number of phonon modes from 5 to 15 THz. The peak of the amorphous perforated silicon film shifts to the left. The peak of amorphous and amorphous perforated films increases when the frequency is less than 5 THz.

**Figure 8:**
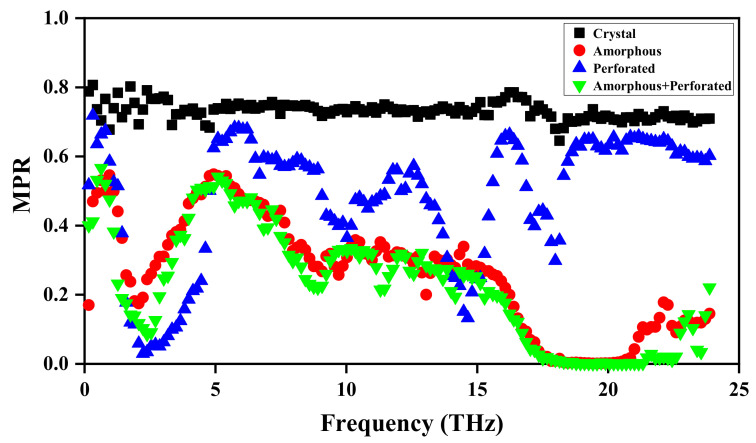
Mode participation ratio of different structures.

The MPR of different structures are shown in Figure 8. For crystalline film, most of the MPR are above 0.6, indicating these phonon modes have been decentered. By contrast, for the film with cloak, most of the MPR are below 0.6, which implies phonon mode localization [52]. Compared with the film with amorphous structure, the degree of localization of phonons in the perforated film is weaker. Compared with the amorphous film, the phonon localization degree of the amorphous perforated film is enhanced, but the degree is not large. This shows that the phonon localization effect produced by the amorphous structure is more obvious than that of the perforated structure. The decrease in MPR leads to the decrease in thermal conductivity, which in turn leads to the generation of cloaking phenomenon. Therefore, the existence of phonon localization in functional region is the main reason for the emergence of the cloaking phenomenon. The more intense the phonon localization, the lower the thermal conductivity, and the more obvious the cloaking effect. The reduction of the vibration frequency of the amorphous silicon atom results in a reduction in the production of phonon, and the holes hinder the transmission of phonon, which ultimately leads to the lowest thermal conductivity of the functional region constructed by the combination of the two methods and the best cloaking effect.

## 4. Conclusions

Nanoscale thermal cloaks have important uses in the field of thermal design to isolate electronic components. In this study, nanoscale thermal cloaks with different structures were constructed by using LAMMPS software. The *RTC* and *T_re_* were used to calculate its performance and the effect to the background temperature field with the existence of the cloak, respectively. Using the phonon localization theory, the cloaking mechanism was explained by calculating the PDOS and the MPR. The conclusions can be summarized as follows:

(1) Compared to the dynamic temperature boundary, the cloaking effect produced under the constant temperature boundary is more obvious. The reason is that the average temperature of the dynamic boundary at the same time is much lower than the temperature of the constant boundary, and the heat flux transferred in the whole system is small, resulting in insignificant cloaking effect.

(2) The ratio of thermal cloaking (*RTC*) is consistent with the calculation result of the response temperature (*T_re_*). For different temperature boundaries, the thermal cloak constructed by amorphous and perforation can produce the best cloaking performance, and at this time, the interference of the external temperature field is minimal.

(3) The phonon localization effect produced by the amorphous structure is more obvious than that of the perforated structure. The decrease in the MPR leads to the decrease in thermal conductivity, which in turn leads to the generation of cloaking phenomenon. Therefore, the existence of phonon localization in the functional region is the main reason for the emergence of the cloaking phenomenon. The more intense the phonon localization, the lower the thermal conductivity, and the more obvious the stealth effect.

There are also some limitations to this work. The degree of disorder in the construction of amorphous structures through the “melting-quenching” technique cannot be measured. We will try to solve this problem in future work.

## Figures and Tables

**Table 1:** *T_re_* under different structures and temperature boundaries.

Structures	Temperature Boundaries	*T_re_*
Amorphous	Constant	0.726
Perforated	Constant	0.873
Amorphous + Perforated	Constant	0.334
Amorphous	Dynamic	0.754
Perforated	Dynamic	0.914
Amorphous + Perforated	Dynamic	0.375

## Data Availability

The data presented in this study are available in this article.
